# Enhanced carotid body chemosensory activity and the cardiovascular alterations induced by intermittent hypoxia

**DOI:** 10.3389/fphys.2014.00468

**Published:** 2014-12-02

**Authors:** Rodrigo Iturriaga, David C. Andrade, Rodrigo Del Rio

**Affiliations:** ^1^Laboratorio de Neurobiología, Departamento de Fisiología, Facultad de Ciencias Biológicas, Pontificia Universidad Católica de ChileSantiago, Chile; ^2^Laboratory of Cardiorespiratory Control, Center of Biomedical Research, Universidad Autónoma de ChileSantiago, Chile

**Keywords:** autonomic dysfunction, carotid body, intermittent hypoxia, hypertension, oxidative stress

## Abstract

The carotid body (CB) plays a main role in the maintenance of the oxygen homeostasis. The hypoxic stimulation of the CB increases the chemosensory discharge, which in turn elicits reflex sympathetic, cardiovascular, and ventilatory adjustments. An exacerbate carotid chemosensory activity has been associated with human sympathetic-mediated diseases such as hypertension, insulin resistance, heart failure, and obstructive sleep apnea (OSA). Indeed, the CB chemosensory discharge becomes tonically hypereactive in experimental models of OSA and heart failure. Chronic intermittent hypoxia (CIH), a main feature of OSA, enhances CB chemosensory baseline discharges in normoxia and in response to hypoxia, inducing sympathetic overactivity and hypertension. Oxidative stress, increased levels of ET-1, Angiotensin II and pro-inflammatory cytokines, along with a reduced production of NO in the CB, have been associated with the enhanced carotid chemosensory activity. In this review, we will discuss new evidence supporting a main role for the CB chemoreceptor in the autonomic and cardiorespiratory alterations induced by intermittent hypoxia, as well as the molecular mechanisms involved in the CB chemosensory potentiation.

## Introduction

The carotid body (CB) located in the bifurcation of the carotid arteries is the main peripheral chemoreceptor sensing arterial levels of PO_2_, PCO_2_, and pH. Also, changes in blood flow, temperature, osmolarity and glucose are able to elicit CB chemosensory excitation (Gonzalez et al., 1994; Pardal and López-Barneo, 2002; Iturriaga and Alcayaga, 2004; Iturriaga et al., 2007). The CB consists of clusters of chemoreceptor (glomus or type I) cells organized around the capillary network, synaptically connected to the nerve terminals of sensory neurons whose somata are in the petrosal ganglion, and surrounded by sustentacular glial (type II) cells. The most accepted model for chemoreception proposes that hypoxia closes K^+^ channels, leading to glomus cell depolarization, entry of Ca^2+^ and the release of excitatory transmitters (ACh and ATP), which in turn increases the discharge in the nerve endings of the chemosensory neurons (Iturriaga and Alcayaga, 2004; Iturriaga et al., 2007). In the last years, new exciting evidences have shown that the CB plays a crucial role in the pathogenesis of several human sympathetic-mediated diseases, including obstructive sleep apnea (OSA), congestive heart failure, resistant hypertension and insulin resistance (Koyama et al., 2000; Prabhakar et al., 2005; Schultz et al., 2007; Iturriaga et al., 2009; Abdala et al., 2012; Del Rio et al., 2013; Paton et al., 2013; Porzionato et al., 2013; Ribeiro et al., 2013). Accordingly, targeting the CB in several pathological conditions has been proposed to be a future promising therapeutic tool for the treatment of sympathetic-mediated diseases. Indeed, the selective ablation of the CB markedly improve rat survival in experimental heart failure (Del Rio et al., 2013), prevent the development of insulin resistance and hypertension in rats fed with high sucrose diet (Ribeiro et al., 2013) and reduced high blood pressure in neurogenic and resistant hypertension (McBryde et al., 2013; Paton et al., 2013).

## Obstructive sleep apnea is an independent risk factor for systemic hypertension

The OSA syndrome elicited by repeated upper airways occlusion, is usually associated with daytime sleepiness, fatigue, and deficits in attention and executive function (Beebe and Gozal, 2002; Idiaquez et al., 2014). Furthermore, OSA is recognized as an independent risk factor for systemic hypertension (~50% of OSA patients develop diurnal hypertension, Somers et al., 2008; Calhoun, 2010), and is associated with stroke, pulmonary hypertension, coronary artery disease and atrial fibrillation (Fletcher, 2000; Parati et al., 2007; Somers et al., 2008; Dempsey et al., 2010). Indeed, several epidemiological studies have shown that OSA is an independent risk factor for the progression of the hypertension, showing a positive relationship between the apnea/hypopnea index (AHI) and high blood pressures (Young et al., 1993; Peppard et al., 2000; Eckert and Malhotra, 2008; Marin et al., 2012). Moreover, results obtained from the Wisconsin Sleep Cohort (an ongoing 21-years longitudinal study performed on 1500 Wisconsin state employees) showed that untreated OSA patients have a high mortality risk associated with AHI (Nieto et al., 2000; Young et al., 2008). According to the “Recommendations for the management of patients with obstructive sleep apnoea and hypertension” recently published by the European Union Cooperation in Scientific and Technological Research Action B26 on OSA, with the endorsement of the European Respiratory Society and the European Society of Hypertension (Parati et al., 2013) OSA is defined as “The combination of at least five obstructive breathing episodes per hour during sleep (apnoea, hypopnoea and respiratory effort related arousal events) and the following diagnostic criteria (A and/or B to be fulfilled). A: Excessive daytime sleepiness that is not better explained by other factors. B: Two or more of the following symptoms that are not better explained by other factors: Choking or gasping during sleep, recurrent awakenings from sleep, unrefreshing sleep, daytime fatigue and impaired concentration.” According to this study, the AHI defines the severity of OSA: mild OSA: AHI 5–15 events/h; moderate OSA: AHI 15–30 events/h and severe OSA: AHI > 30 events/h (Parati et al., 2013).

## Pathophysiological mechanisms of OSA-induced hypertension

The cyclic obstruction of the upper airways during OSA leads to intermittent hypoxia and hypercapnia, negative intrathoraxic pressure, sleep fragmentation, and micro-arousals (Somers et al., 2008; Dempsey et al., 2010). During the airway occlusion, the resulting hypoxia and hypercapnia stimulates the CB chemoreceptor eliciting reflex acute sympathetic, hypertensive and hyperventilatory responses (Gozal and Kheirandish-Gozal, 2008; Somers et al., 2008; Garvey et al., 2009; Dempsey et al., 2010). Among these disturbances, the chronic intermittent hypoxia (CIH) is considered the main factor for the development of diurnal hypertension (Lavie, 2003; Gozal and Kheirandish-Gozal, 2008; Lévy et al., 2008; Somers et al., 2008; Arnardottir et al., 2009; Dempsey et al., 2010). Although the link between OSA and hypertension is well proved, the mechanisms underlying the pathogenesis of the hypertension are not entirely known. The most accepted proposal states that CIH elicits systemic oxidative stress, inflammation, and sympathetic hyperactivity, which led to endothelial dysfunction and the hypertension (Lavie, 2003; Somers et al., 2008; Garvey et al., 2009; Ryan et al., 2009; Dempsey et al., 2010). Nevertheless, conclusions from studies performed in OSA patients are controversial, because OSA patients often present concomitant morbidities (i.e., obesity and metabolic alterations), which are confounding factors that increase the cardiovascular risk. Thus, animal model of CIH, which simulates the hypoxic-reoxygenation episodes and reproduce several cardiovascular pathologic features of OSA including sympathetic hyperactivity and hypertension, are the gold-standard model to study mechanisms involved in OSA (Fletcher et al., 1992; Peng et al., 2003, 2011; Iturriaga et al., 2005, 2009; Prabhakar et al., 2005; Schulz et al., 2008; Dematteis et al., 2009; Del Rio et al., 2010, 2011a, 2012; Dumitrascu et al., 2013).

OSA produces sympathetic hyperactivity, demonstrated by an increased muscle sympathetic neural activity to blood vessels (Carlson et al., 1993) and excessive accumulation of urinary catecholamines (Dimsdale et al., 1995). Similarly, animals exposed to CIH show enhanced sympathetic responses to hypoxia, and develop systemic hypertension (Fletcher et al., 1992; Greenberg et al., 1999; Dick et al., 2007; Feng et al., 2008; Huang et al., 2009; Zoccal et al., 2009; Del Rio et al., 2010; Marcus et al., 2010). The autonomic dysfunction is characterized by enhanced sympathetic outflow, a reduction of the efficiency of the cardiac baroreflex sensitivity and alterations of heart rate variability (HRV). Indeed, non-invasive spectral analysis of HRV shows an increased ratio of low (LF) to high frequency (HF) band power, with a relative predominance of the LF band and a reduced contribution of the HF band, suggesting preponderance of the sympathetic drive in patients with OSA (Narkiewicz et al., 1998a; Shiomi et al., 1996) and animals exposed to CIH (Lai et al., 2006; Rey et al., 2008; Del Rio et al., 2010). Furthermore, it has been shown that CIH elicits vagal withdrawal, attributed in part to neuronal loss in ambiguous nucleus (Yan et al., 2008). Therefore, it is likely that the enhanced sympathetic to parasympathetic balance along with the reduction of the baroreflex could contribute to impair HRV and the regulation of vasomotor tone of blood vessels finally eliciting systemic hypertension.

In addition, OSA syndrome is also associated with endothelial dysfunction and vascular remodeling (Ip et al., 2004; Patt et al., 2010). OSA patients show an increased intima-media thickness (Minoguchi et al., 2005; Monneret et al., 2012) and a reduced nitric oxide-mediated vasodilatation (Kato et al., 2000). Similarly, some studies found that CIH reduced acetylcholine (ACh)-mediated vasodilation in rats (Tahawi et al., 2001; Dopp et al., 2011), but other reported a normal endothelial function in hypertensive CIH-treated rats (Julien et al., 2003; Lefebvre et al., 2006). Indeed, Lefebvre et al. (2006) found that CIH had no effect on the ACh-mediated vasodilatation of carotid, aortic and mesenteric beds, as well as on the contractile responses induced by noradrenaline and angiotensin II (Ang II) in arteries from CIH-rats compared to the arteries from control rats. However, they found that the contraction induced by endothelin-1 (ET-1) was higher in arteries from CIH-rats. More recently, Philippi et al. (2010) studied the time-course of the alteration of the endothelium dependent vasodilation in rats exposed to CIH. They found that CIH produces functional and structural changes in skeletal muscle arteries within the first 2 weeks of CIH, and those alterations were accompanied by systemic oxidative stress. Friedman et al. (2014) found that ROS generation during CIH activates NFATc3, which in turn increase the vascular response to ET-1. The administration of Tempol, a superoxide dismutase (SOD) mimetic, during CIH prevents the increased NFATc3 activity in the arteries from CIH-exposed mice, supporting that ROS is an important upstream signal in the CIH-induced NFATc3. Together, the available information suggest that vascular beds are affected by exposure to CIH, and that enhanced contractile responsiveness to vasoactive molecules such as ET-1 is critically dependent on ROS formation.

## Intermittent hypoxia enhances CB chemosensory discharges in normoxia and hypoxia

Patients recently diagnosed with OSA, present potentiated pressor and ventilatory responses to hypoxia (Narkiewicz et al., 1998a,b, 1999), suggesting that the peripheral hypoxic chemoreflex were enhanced by CIH. Fletcher et al. (1992) were the first to obtain evidences that the CB is involved in the hypertension induced by CIH. They found that the bilateral CB denervation prevented the development of hypertension in rats exposed to CIH for 35 days. Despite this seminal observation, the proposal that the CB contributes to the progression of the cardiovascular pathologies associated to OSA was not seriously considered. However, in the last decade a growing body of new evidences have support the proposal that the CB contributes to the progression of the CIH-induced hypertension (See for reviews: Prabhakar et al., 2005; Smith and Pacchia, 2007; Weiss et al., 2007; Somers et al., 2008; Garvey et al., 2009; Iturriaga et al., 2009; Dempsey et al., 2010). Recordings of rat and cat CB chemosensory discharges *in situ* and *in vitro* have demonstrate that CIH selectively increases basal chemosensory discharges in normoxia, and potentiates chemosensory and ventilatory responses to acute hypoxia (Peng et al., 2003, 2004; Rey et al., 2004, 2006; Prabhakar et al., 2005; Iturriaga et al., 2009; Del Rio et al., 2010, 2012). In addition, CIH induces plasticity of the CB chemosensory activity manifested as long-term facilitation. Indeed, Peng et al. (2003) found that chemosensory baseline discharges increased when the CB was excited by repetitive acute intermittent hypoxia in rats exposed to CIH. They reported that following 10 episodes of 12% O_2_ lasting for 15 s, interspersed with 5 min of 95% O_2_, the baseline chemosensory discharge increased with each episode of hypoxia, which persist for 60 min following the end of the hypoxic stimulus.

The mechanisms underlying the enhanced CB chemosensory reactivity to hypoxia induced by CIH are not entirely known (Iturriaga et al., 2009). Oxidative stress (Peng et al., 2003, 2009; Del Rio et al., 2010, 2012; Marcus et al., 2010), ET-1 (Rey et al., 2006, 2007; Pawar et al., 2009), Ang II (Lam et al., 2008, 2012; Fung, 2014), and pro-inflammatory cytokines (Iturriaga et al., 2009; Del Rio et al., 2011a, 2012; Lam et al., 2012) have been associated with the CB chemosensory potentiation. However, the primary molecular target responsible for the increased chemoreceptor discharge remains unknown. Recently, we studied the effects evoked by CIH on TASK K^+^ channel activity and the depolarization induced by acute hypoxia in CB glomus cells from adult rats exposed to CIH (Ortiz et al., 2013). We measured membrane potential, single channel and macroscopic currents in the presence of TEA and 4-aminopyridine in CB chemoreceptor cells isolated from adult rats exposed to CIH for 7 days. CIH treatment did not change the resting membrane potential, but the hypoxic-evoked depolarization increased by 2-fold. Moreover, the hypoxic inhibition of the open probability of the TASK-K^+^ channel was larger and faster in glomus cells from CIH-treated rats. This novel effect of CIH may contribute to explain the potentiation of CB oxygen chemoreception.

## Molecular mechanisms underlying enhanced carotid body chemosensory activity during intermittent hypoxia

### Oxidative stress contributes to enhance the carotid chemosensory activity during intermittent hypoxia

ROS and reactive nitrogen species (RNS) have been proposed as mediators of the cardiovascular alterations in OSA patients (Christou et al., 2003; Lavie, 2003; Gozal and Kheirandish-Gozal, 2008; Jelic et al., 2008; Lévy et al., 2008) and animal exposed to CIH (Peng et al., 2003, 2009, 2011; Chen et al., 2005; Troncoso Brindeiro et al., 2007; Huang et al., 2009; Del Rio et al., 2010, 2012). Studies performed in OSA patients and animals exposed to CIH have shown that hypoxia-reoxygenation produces systemic oxidative stress due to the accumulation of ROS and RNS. Peng et al. (2003) proposed that superoxide radical participates in the potentiation of the rat CB chemosensory responses to hypoxia induced by CIH. They found that pre-treatment of rats for 10 days before and concomitant with the exposure to CIH with manganese (III) tetrakis (1-methyl-4-pyridyl) porphyrin pentachloride (MnTMPyP), a SOD mimetic, prevents the CB chemosensory potentiation. In addition, they found that CIH decreases the activity of the aconitase enzyme in the CB and the activity of the complex I of the mitochondrial electron transport chain, suggesting that the mitochondria function is affected by CIH and represent a potential source of ROS production (Peng et al., 2003). In addition, Peng et al. (2009) found that acute hypoxia produced a larger increase in NOX activity in CBs from rats exposed to CIH for 10 days compared to the NOX activity found in control CBs, suggesting that NADPH oxidase contributes to generate ROS during CIH. Recently, Schulz et al. (2014) have shown that NADPH oxidase 2 (NOX2) knockout blocks the development of the hypertension induced by CIH. Indeed, they found that mice showed significant arterial blood pressure elevations after CIH. The hypertension was attenuated by l inhibition of NOX by apocynin, whereas NOX2 was not upregulated in the heart, aorta, and femoral and carotid arteries of CIH-mice. Therefore, they suggested that the CIH-induced arterial hypertension is mediated by ROS derived from an activation of NOX2 within cells located outside the cardiovascular system.

We studied the role of nitro-oxidative stress on the enhanced CB chemosensory function and hypertension in rats exposed to CIH for 21 days (Del Rio et al., 2010). We measured 3-nitrotyrosine (3-NT) formation in the CB as an index of oxidative stress. Superoxide reacts with NO to generate peroxynitrite, a powerful oxidizing agent that nitrates protein tyrosine-residues forming 3-NT. We found that CIH increased plasma lipid peroxidation and the formation of 3-NT in the CB. In addition, CIH enhanced the CB chemosensory and ventilatory responses to acute hypoxia, alters HRV and elicits hypertension. Concomitant administration of ascorbic acid reduced the increased systemic and local CB nitro-oxidative stress, the potentiation of CB chemosensory and ventilatory responses to hypoxia, as well as the hypertension in rats exposed to CIH (Del Rio et al., 2010). These results agree and extend previous observations that antioxidant treatment prevented the CB chemosensory potentiation (Peng et al., 2003) and the hypertension (Troncoso Brindeiro et al., 2007) in rats exposed to CIH.

**Figure 1:**
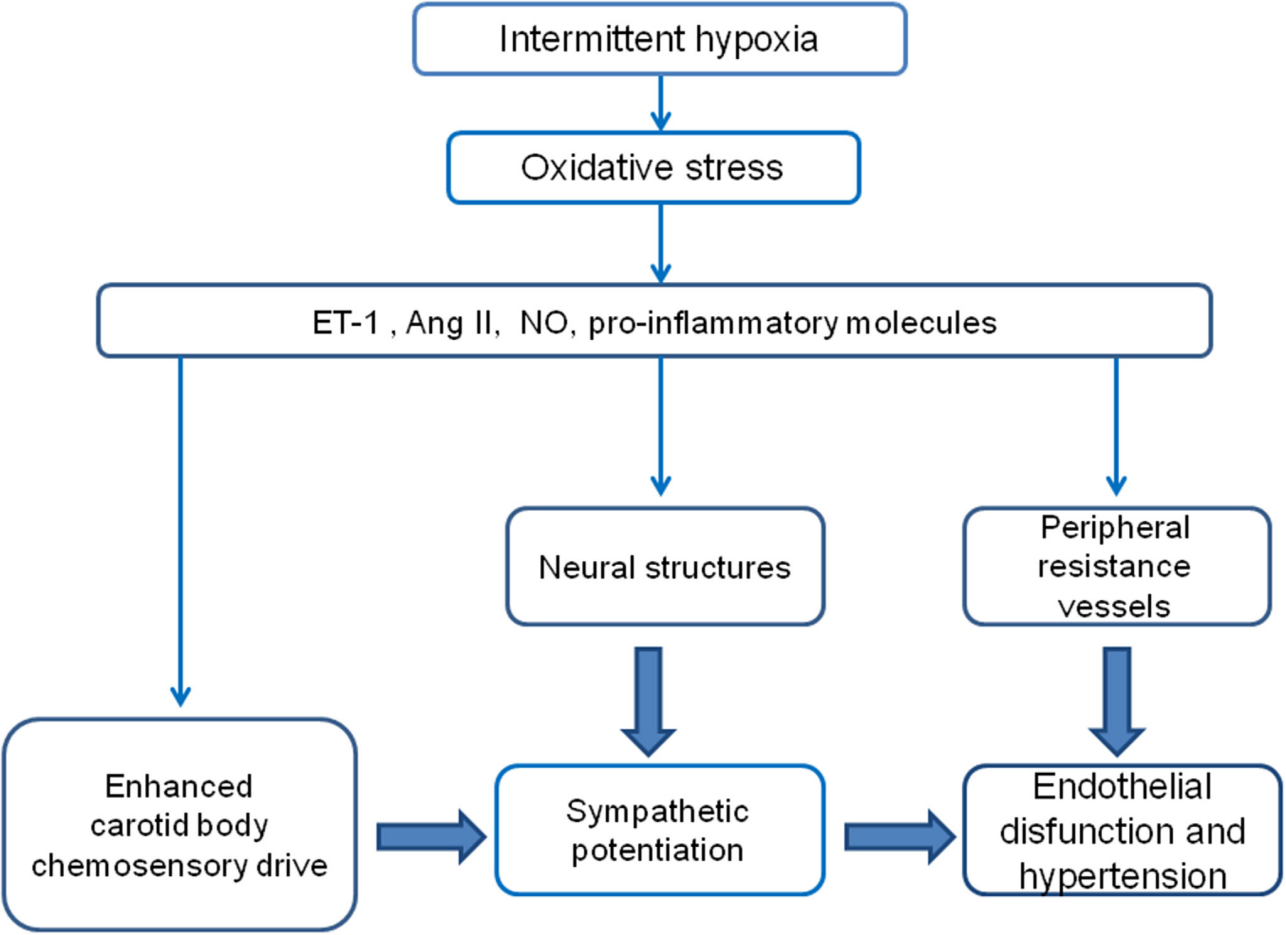
**Diagram of the proposed hypothetical mechanisms involved in the potentiation of the CB chemosensory response to hypoxia and the development of hypertension induced by CIH**. It is likely that the hypoxic-reoxygenation cycles enhance the CB chemosensitivity to hypoxia, which in turn contributes to elicit a persistent augmented sympathetic neural drive.

The available evidence indicates that oxidative stress is involved in the CIH-induced CB potentiation, but it is matter of debate whether ROS are the primary signal, because ROS *per se* do not increase the CB chemosensory discharges. Indeed, H_2_O_2_ does not increase the carotid chemosensory discharge in rats (Peng et al., 2009) or cats CB (Osanai et al., 1997). In addition, modification of ROS production in rat glomus cells did not alter the catecholamine secretion, suggesting a lack of a causal link between ROS and glomus cells excitability (Gonzalez et al., 2007). Thus, it is possible that other molecules activated by the oxidative stress mediate the enhancing effects of CIH on CB oxygen chemoreception (See Figure 1 and Table 1).

**Table 1:** **Possible mediator of the CIH effects on CB chemosensory potentiation**.

**Mediator**	**References**
Endothelin 1	Rey et al., 2006, 2007; Iturriaga, 2013
Endothelin-1 (dependent on ROS)	Pawar et al., 2009; Peng et al., 2013
Reduced NO production (reduced nNOS and eNOS-ir levels).	Marcus et al., 2010; Del Rio et al., 2011a; Moya et al., 2012
Angiotensin II (dependent on O_2_- production signaling through AT1 receptor)	Lam et al., 2008, 2012; Marcus et al., 2010; Peng et al., 2011; Fung, 2014
Pro-inflammatory cytokines	Iturriaga et al., 2009; Del Rio et al., 2011b, 2012; Lam et al., 2012

### Endothelin-1

We and other have proposed that ET-1 is involved in the potentiation of the CB chemosensory discharge induced by CIH (Rey et al., 2006, 2007; Pawar et al., 2009; Iturriaga, 2013; Peng et al., 2013) and in the development of hypertension (Troncoso Brindeiro et al., 2007; Allahdadi et al., 2008). Rey et al. (2006) found that CIH increased 10-times the ET-1 immunoreactivity in endothelial, smooth muscle and glomus cells from CBs from cats exposed to CIH for 4 days, without changes in ET-1 plasma concentration. ET-1 elicits chemosensory excitation in both *in situ* and *in vitro* perfused cat CB preparation, but not in the superfused CB preparation, showing a predominant vascular effect. The CIH-induced potentiation of baseline discharges and hypoxic chemosensory responses in the perfused cat CB preparation was reduced by the unspecific ET-1 receptor blocker bosentan (Rey et al., 2006). These results suggest that a local increase of ET-1 in the CB may contribute to enhance the CB chemosensory tone induced by CIH, through a predominant vasomotor mechanism. Pawar et al. (2009) found that CIH enhanced the basal release of ET-1 and produces upregulation of the ET-A receptor, while the administration of MnTMPyP, which prevent the oxidative stress, reduced the increased release of ET-1 and the enhanced CB chemosensory responses to hypoxia. In the same way, the concurrent treatment with the ET-A receptor inhibitor BQ-123 prevented the development of the hypertension in rats exposed to CIH for 14 days (Allahdadi et al., 2008). Thus, ET-1 seems to be involved in the enhanced hypoxic CB chemosensory responses and in the progression of the hypertension following CIH. More recently, Peng et al. (2013) found that CIH increased the activity of the endothelin converting enzyme (ECE), which paralleled the raise of the ET-1 level in the neonatal rat CB. Since MnTMPyP prevented these effects, they proposed that oxidative stress was involved in the increased ET-1 expression. In addition, they found that hypoxia facilitates ET-1 release from CIH-treated CB, but not from control rat CB. These results support that a ROS-dependent release of ET-1, which activates the ET-A receptor is involved in the potentiation of the CB chemosensory responses to hypoxia elicited by CIH in neonatal rats. However, it is worth to note that Del Rio et al. (2011a) and Lam et al. (2006) found that CIH transiently increases the levels of ET-1 in the adult rat CB during the first week of CIH, but later ET-1 levels returned to the control levels, suggesting that ET-1 may contribute to the enhanced CB responsiveness to hypoxia in the early phase of CIH.

### Nitric oxide

We studied the changes in the expression of eNOS in the CB, along with the progression of potentiated CB chemosensory responses to hypoxia in rats exposed to CIH for 7 to 21 days (Del Rio et al., 2011a). Exposure to CIH for 7 days enhanced CB chemosensory responses to hypoxia and produced a significant decrease in the eNOS immunoreactivity in the CB, which persisted for 21 days of CIH, suggesting that CIH may decrease the NO levels in the CB. Thus, we measured NO production—via nitrite generation in the incubation medium—from rat CBs exposed to CIH, and found a reduction in the NO production after 7 days of CIH that correlates with the reduced eNOS expression (Del Rio et al., 2011a; Moya et al., 2012). Since NO is an inhibitory modulator of CB chemosensory discharges, we hypothesized that a reduced NO level may contribute to enhance the basal CB discharges and the chemosensory responses to hypoxia (Moya et al., 2012). This interpretation is supported by the finding of Marcus et al. (2010), showing that CIH decreased the expression of the nNOS in the rat CB, suggesting that the removal of the normal inhibitory NO influence contributes to enhancing the CB chemosensory responses to hypoxia. We found a marked increase of 3-NT in the CB from rats exposed to CIH, which correlates with the enhanced chemosensory responses to hypoxia (Del Rio et al., 2011a), supporting the idea that oxidative-nitrosative stress plays a critical role in CB chemosensory potentiation induced by CIH (Iturriaga et al., 2009; Del Rio et al., 2010). Thus, the available data suggests that peroxynitrite formation due to the reaction of NO with the superoxide radical is a critical step in the CB chemosensory potentiation induced by CIH (Del Rio et al., 2010, 2011a).

### Angiotensin II

The role of Angiotensin II on the enhanced CB chemosensory responses induced by CIH has been extensively reviewed by Fung (2014). The CB constitutively expresses the renin-angiotensin system (RAS), and responds to Ang II due to the functional AT-1 receptor expression in the CB glomus cells (Fung et al., 2001). Lam et al. (2014) found that CIH increased the expression of angiotensinogen and AT1 receptor in the rat CB glomus cells. They also found that the elevation of intracellular Ca^2+^ in response to exogenous Ang II was enhanced in glomus cells from CIH-rats. The pretreatment with losartan abolished the Ang II-induced Ca^2+^ response, suggesting an involvement of AT1 receptors, and attenuated the levels of gp91 (phox) and macrophage infiltration in the CB. Thus, the unregulated RAS expression may play a role in the enhanced CB chemosensory activity and local inflammation via AT1 receptor activation during CIH.

### Pro-inflammatory cytokines

Among the molecules up regulated in the CB by CIH, such as ET-1, Ang II, VEGF and iNOS (Rey et al., 2006, 2007; Lam et al., 2008, 2012, 2014; Del Rio et al., 2010, 2011a,b), pro-inflammatory cytokines have been proposed as mediators of the CB chemosensory potentiation induced by CIH (Lam et al., 2008; Iturriaga et al., 2009; Del Rio et al., 2011a, 2012) and cardiovascular pathologies in OSA patients (Vgontzas et al., 2004; Minoguchi et al., 2005; Biltagi et al., 2008; Ryan et al., 2009). Accordingly, we studied the time-course of the changes in the immunohistological levels of TNF-α, IL-1β, and IL-6 in the CB, along with the progression of the enhanced CB chemosensory responses to hypoxia in rats exposed to CIH for 7 to 21 days (Del Rio et al., 2011a). We found that CIH progressively increases the levels of TNF-α and IL-1β in the rat CB without modifying their plasma levels. On the contrary, Lam et al. (2012) reported that exposure of rats to intermittent hypoxia for 7 days increases the levels of IL-1β, TNF-α, and IL-6 in the CB, and found macrophage infiltration, which was reduced by daily treatment with the anti-inflammatory drugs dexamethasone or ibuprofen. Oxidative stress increases the synthesis of pro-inflammatory cytokines, mediated by the activation of the transcriptional factors NF-κB, activator protein 1 and HIF-1α (Prabhakar and Semenza, 2012). In response to oxidative stress, it is known that HIF-1α produced the translocation of NF-κB to the nucleus augmenting the expression of pro-inflammatory genes such as IL-1β, TNF-α, and ET-1 (Reuter et al., 2010). Accordingly, we found that CBs from rats exposed to CIH for 21 days showed higher levels of the p65 sub-unit of NF-κB suggesting a plausible role for this factor in the upregulation of the pro-inflammatory cytokines during CIH (Del Rio et al., 2012). We tested the hypothesis that CIH induced a ROS-dependent increased TNF-α and IL-1β levels in the CB, which may contribute to the CB chemosensory potentiation (Del Rio et al., 2012). Accordingly, we studied the effects of ibuprofen on TNF-α and IL-1β levels in the rat CB, the potentiation of the CB chemosensory and ventilatory hypoxic responses and the development of systemic hypertension (Del Rio et al., 2012). Ibuprofen prevented the overexpression of the cytokines, the enhanced hypoxic ventilatory response and the hypertension, but failed to block the enhanced CB chemosensory responses. Thus, our studies suggest that the upregulation of TNF-α and IL-1β in the CB induced by CIH is linked to oxidative stress, as well as the enhanced CB chemosensory responsiveness to hypoxia, but the chemosensory potentiation does not depend on the increased TNF-α and IL-1β levels in the CB. However, pro-inflammatory cytokines contribute to enhance the hypoxic ventilatory response and the hypertension induced by CIH, suggesting that multiple mechanisms may participate in the cardiorespiratory alterations induced by CIH.

## Contribution of central cardiorespiratory centers and arterial vessels to the hypertension induced by CIH

The sympathetic hyperactivity induced by CIH is likely to be the result of the enhanced CB chemosensory drive, but we cannot preclude excitatory effects of CIH on other structures of the chemorefelex pathway. Indeed, the same molecules that are involved in the enhanced CB chemosensitivity (e.g., Ang II, ET-1, and NO) could act at multiple sites to contribute to CIH-induced arterial blood pressure rise (e.g., higher CNS centers, peripheral arteries vessels). The chemosensory petrosal neurons that innervate the CB glomus cells project to the NTS in the brainstem, which is the main integrative nucleus for visceral inputs. The NTS send projections to the RVLM that contain the pre-sympathetic neurons projecting to the pre-ganglionar neurons in the spinal cord. RVLM neurons participate in the control of BP, and in the CB-mediated activation of the sympathetic responses (Guyenet et al., 2010). It has been shown that CIH increased the expression of the neuronal activation markers c-Fos, and FosB/ΔFos in the NTS and RVLM. Indeed, Greenberg et al. (1999) found that CIH-exposure of rats for 30 days increased c-fos labeling in the NTS and the RVLM. More recently, several studies reported that CIH increases FosB/ΔFosB in the subfornical organ, the median preoptic nucleus, the paraventricular nucleus, the NTS and the RVLM (Knight et al., 2011; Cunningham et al., 2012; Bathina et al., 2013). Thus, other structures outside the brainstem might contribute to intermittent hypoxia-induced hypertension (e.g., paraventricular nucleus of the hypothalamus, as shown by Sharpe et al., 2013). The available evidences strongly suggests that oxidative stress is the key mediator of the enhanced CB chemosensory responses to hypoxia and the hypertension induced by CIH, but the actions of the oxidative stress on the BP regulation in rats exposed to CIH may occur in multiple sites of the chemoreflex pathway, including the NTS, RVLM, and/or the arterial blood vessels. Indeed, it has been proposed that superoxide anions in the brainstem contribute to elevate the arterial blood pressure in rat models of neurogenic hypertension such as the stroke-prone spontaneously hypertensive rat (Kishi et al., 2004) and Ang II induced hypertension (Chan and Chan, 2012). Although it is well known that oxidative stress, produced by Ang II and NADPH activation, in the brainstem elicits sympathetic activation, the role played by the oxidative stress induced by CIH in the progression of the hypertension is less known. In addition, Marcus et al. (2012) found that CIH impairs the vasodilatory responses in small arteries isolated from the skeletal muscle circulation in rats, an effect blocked by losartan, a Ang II type 1 receptor blocker. Intermittent hypoxia also caused an increase in the ratio of Ang II type 1 receptors (responsible for vasoconstriction and trophic effects) to Ang II type 2 receptors (responsible for vasodilation and anti-trophic properties) in peripheral arteries. On the other hand, oxidative stress has also been involved in the impaired vasodilatation in response to ACh in rats exposed to CIH. Indeed, the treatment of CIH-exposed rats with Tempol restores the normal vascular function (Phillips et al., 2006). Moreover, Dopp et al. (2011) reported that concomitant treatment with allopurinol, a xanthine oxidase inhibitor, attenuated the impairment of ACh induced vasodilatation in gracillis arteries of rats exposed to CIH for 14 days.

## Conclusions and future directions

The pathophysiological mechanisms involved in the development of hypertension in OSA are not fully understood. It is widely accepted that the CIH-induced oxidative stress contributes to enhance the CB chemosensory reactivity to oxygen and to the progression of the hypertension (Figure 1). Several studies have shown that concomitant administration of antioxidants, SOD mimetic, anti-inflammatory agents, ETA, and AT-1 receptor blockers, all of them reducing the levels of ROS formation and/or blocking the downstream signaling pathways induced by CIH, effectively prevents the enhanced CB chemosensory as well as the development of the hypertension. In addition, results showing that ablation of the CBs before the exposure to CIH significantly prevent the development of the hypertension strongly suggest a main role of the CB in the progression of the hypertension following CIH. However, the effect of the oxidative stress on the arterial blood pressure in rats exposed to CIH may also occur in multiple sites of the chemoreflex pathway, including the CB, the central cardiorespiratory centers and/or the arterial vessels. Thus, understanding how the oxidative stress and the molecules activated by CIH may interact at the CB and systemic levels would provide insights into the generation of the cardiovascular complications of OSA.

### Conflict of interest statement

The authors declare that the research was conducted in the absence of any commercial or financial relationships that could be construed as a potential conflict of interest.
